# In vitro analysis of the segmental flexibility of the thoracic spine

**DOI:** 10.1371/journal.pone.0177823

**Published:** 2017-05-16

**Authors:** Hans-Joachim Wilke, Andrea Herkommer, Karin Werner, Christian Liebsch

**Affiliations:** Institute of Orthopaedic Research and Biomechanics, Trauma Research Centre Ulm, University of Ulm, Ulm, Germany; Rush University Medical Center, UNITED STATES

## Abstract

Basic knowledge about the thoracic spinal flexibility is limited and to the authors’ knowledge, no in vitro studies have examined the flexibility of every thoracic spinal segment under standardized experimental conditions using pure moments. In our in vitro study, 68 human thoracic functional spinal units including the costovertebral joints (at least n = 6 functional spinal units per segment from T1-T2 to T11-T12) were loaded with pure moments of ±7.5 Nm in flexion/extension, lateral bending, and axial rotation in a custom-built spine tester to analyze range of motion (ROM) and neutral zone (NZ). ROM and NZ showed symmetric motion behavior in all loading planes. In each loading direction, the segment T1-T2 exhibited the highest ROM. In flexion/extension, the whole thoracic region, with exception of T1-T2 (14°), had an average ROM between 6° and 8°. In lateral bending, the upper thoracic region (T1-T7) was, with an average ROM between 10° and 12°, more flexible than the lower thoracic region (T7-T12) with an average ROM between 8° and 9°. In axial rotation, the thoracic region offered the highest overall flexibility with an average ROM between 10° and 12° in the upper and middle thoracic spine (T1-T10) and between 7° and 8° in the lower thoracic spine (T10-T12), while a trend of continuous decrease of ROM could be observed in the lower thoracic region (T7-T12). Comparing these ROM values with those in literature, they agree that ROM is lowest in flexion/extension and highest in axial rotation, as well as decreasing in the lower segments in axial rotation. Differences were found in flexion/extension and lateral bending in the lower segments, where, in contrast to the literature, no increase of the ROM from superior to inferior segments was found. The data of this in vitro study could be used for the validation of numerical models and the design of further in vitro studies of the thoracic spine without the rib cage, the verification of animal models, as well as the interpretation of already published human in vitro data.

## Introduction

Few studies have focused on the biomechanics of the thoracic spine, because prior research mainly focused on the lumbar [1–4] and the cervical spine [5–7]. This is due to the fact that chronic degenerative diseases of the thoracic spine are reported to have a lower incidence and therefore seem to be clinically of lower importance [8]. However, pathologies of the thoracic spine should not be neglected. The overall number of vertebral fractures, for instance, is, in absolute terms, just slightly higher in the lumbar spine than in the thoracic spine [9]. Furthermore, the growing number of traffic and sports accidents lead to an increasing number of serious injuries, particularly in the lower thoracic spinal segment [10]. These fractures are very likely associated with neurological complications, since the thoracic spinal canal is quite narrow and high forces are required for the formation of vertebral fractures due to the stabilizing effect of the rib cage. Also complex osteoporotic vertebral fractures and spinal metastases are indications for reconstructive and stabilizing surgeries in the thoracic spine; their number has steadily increased in the recent past due to demographic changes [11, 12]. To achieve optimum restoration of injured thoracic spinal structures, basic knowledge of the biomechanics of the intact thoracic spine is mandatory.

Finite element models provide a useful tool for the analysis of several spinal diseases, since they offer the possibility of performing detailed studies of various biomechanical variables and allow individualized simulations of surgical correction procedures [13–17]. For the calibration and validation of finite element models of the healthy spine, a comprehensive database including experimental in vivo and in vitro biomechanical data of asymptomatic thoracic spines is required [18, 19]. However, the limited available data of previous studies with partially contradictory data and the variety of measurement methods illustrate the need for current basic research using new standardized measurement techniques.

Biomechanical investigations regarding the thoracic spinal flexibility are scarce. The first comprehensive in vitro study on thoracic spine flexibility was carried out by White in 1969 [20]. In this biomechanical study, a two-dimensional analysis of monosegmental specimens and a three-dimensional analysis of polysegmental specimens were performed. So far, this study represents the basic knowledge of thoracic spine flexibility. However, the loads were not applied by means of pure moments in this study, while the application of pure moments is the gold standard in spine biomechanics today [21, 22]. A subsequent in vitro study of Panjabi et al. used pure moments to determine the mechanical properties of the thoracic spine by using load-deformation curves and calculating flexibility coefficients. They performed exemplary measurements, each with n = 1 monosegmental specimen for the whole thoracic region from T1-T2 to T11-T12 [23]. Single functional spinal units of the thoracolumbar junction were investigated by Markolf et al. and Oxland et al. [24, 25]. In all these in vitro studies pure moments were applied, but different measurement equipment and torque levels were used. In 1990, White and Panjabi gave an overview of the ranges of motion for all functional spinal units of the human spine. Multiple in vivo and in vitro studies, performed by different authors, were combined in this overview, leading to high variations within the data [26].

The aim of the present study was therefore to investigate the segmental range of motion and neutral zone of the healthy human thoracic spine by applying pure moments, with a sufficient number of specimens, under controlled, standardized testing conditions.

## Materials and methods

A total of 68 thoracic functional spinal units (FSUs) from 29 human donors were tested. For each of the eleven segmental levels of T1-T2 to T11-T12, n = 6 specimens were used for testing, except for the levels T4-T5 and T7-T8, of which each n = 7 specimens were available. The average age of the donors was 57 years (40–80 years), whereby 13 of the donors were male and 16 female (Table 1). None of the specimens showed any visible ligamentous, discogenic, or bony damage relevant to biomechanical testing. Tumorous or fracture related damages were excluded prior to preparation using conventional X-ray images (Faxitron 43805N, Hewlett Packard, Palo Alto, USA). The spines were stored at -20°C. Prior to testing, the specimens were thawed at 4°C and prepared at room temperature. Muscles, nerves, fat, and other soft tissues were carefully dissected while preserving the ligaments, joint capsules, intervertebral discs, and costovertebral joints. The ribs were shortened to a length of about 1.5 cm using a saw.

**Table 1:** Donor age (in years) and sex (m = male, f = female) of the thoracic FSUs.

Segmental level	#1	#2	#3	#4	#5	#6	#7	Mean ± SD
T1-T2	53, f	58, f	46, f	40, m	43, f	60, m	-	50 ± 8
T2-T3	57, f	56, m	79, m	66, f	46, m	58, m	-	60 ± 10
T3-T4	54, m	45, m	53, f	59, f	58, f	60, m	-	55 ± 5
T4-T5	54, f	57, f	44, f	66, f	46, f	63, f	56, m	55 ± 7
T5-T6	54, m	71, f	53, f	58, f	60, m	80, f	-	63 ± 10
T6-T7	54, f	44, f	59, m	43, f	46, f	56, m	-	50 ± 6
T7-T8	76, f	71, f	53, f	58, f	60, m	65, m	66, f	64 ± 7
T8-T9	56, m	54, f	46, f	46, m	51, m	54, m	-	51 ± 4
T9-T10	50, m	57, f	71, f	62, f	75, f	65, m	-	63 ± 8
T10-T11	54, m	69, m	60, m	51, m	63, f	44, f	-	57 ± 8
T11-T12	49, f	71, f	62, f	59, m	65, m	57, f	-	61 ± 7

The upper half of the cranial vertebra and the lower half of the caudal vertebra were embedded in polymethylmethacrylate (PMMA, Technovit 3040, Heraeus Kulzer, Wehrheim, Germany). Before embedding, screws were fixed in the cranial and caudal endplates of each FSU to ensure a firm connection between the vertebral bodies and the subsequent plastic cast. The disc was adjusted horizontally and the costovertebral joints, the facet joints, as well as the inter- and supraspinous ligaments were covered with modelling clay during embedding to preserve the full mobility of the FSUs. After embedding, flanges were coaxially fixed to the PMMA blocks. During preparation and testing, the specimens were kept moist with physiological saline (0.9%) [21].

**Fig 1:**
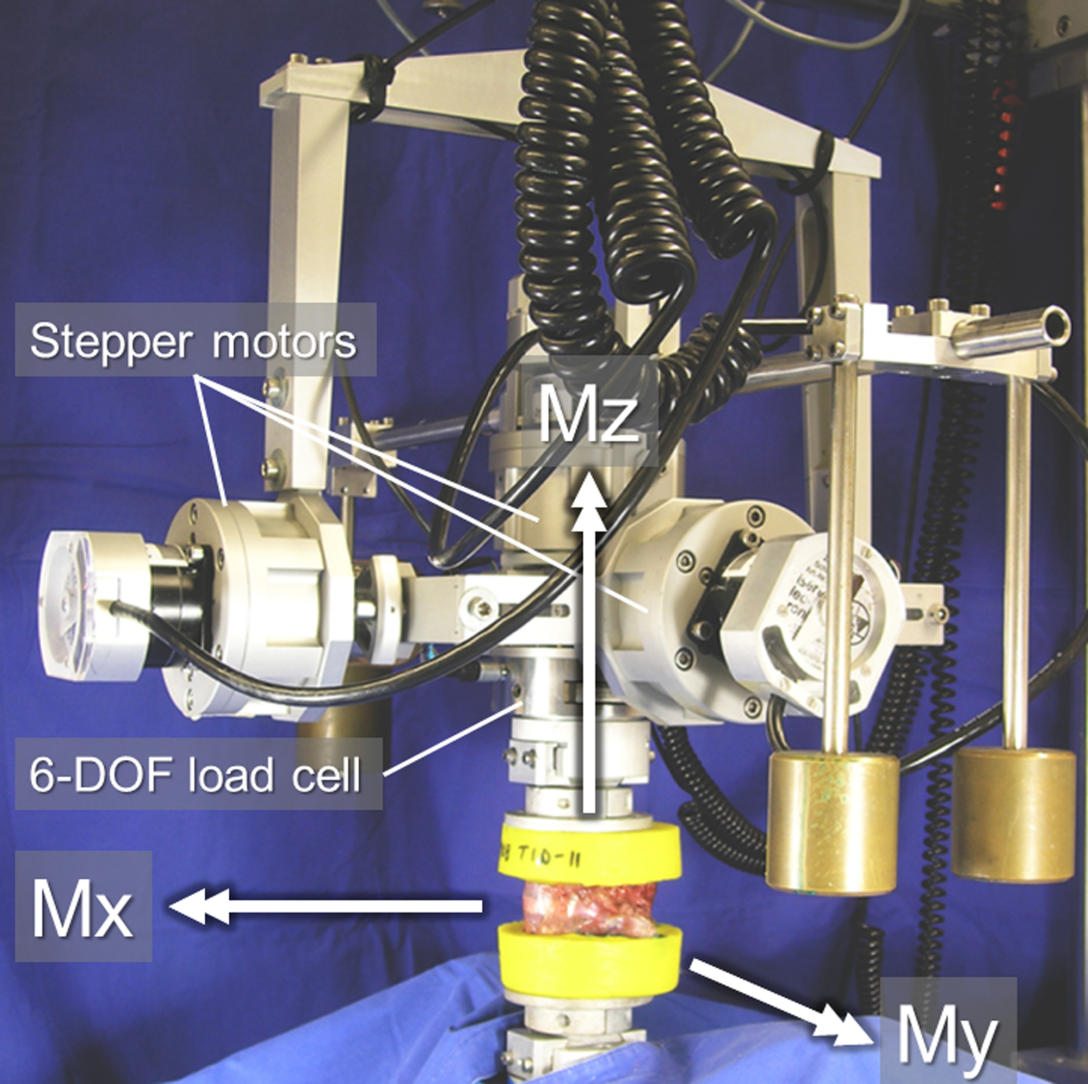
Experimental setup. A typical thoracic spinal motion segment before load application in the spine tester.

After manual alignment regarding the anatomical planes, the 68 FSUs were each loaded in a custom-built spine tester (Fig 1) by applying pure moments of ±7.5 Nm in lateral bending (±Mx), flexion/extension (±My), and axial rotation (±Mz) [27]. While the monitored rotation axis was engaged, the specimens were allowed to move almost unconstrained in the remaining five degrees of freedom due to a traveling gantry and balancing weights (RMS errors in maximum off axis torques: Mx = 0.2 Nm, My = 0.2 Nm, Mz = 0.1 Nm, RMS errors in maximum off axis forces: Fx = 6.2 N, Fy = 7.0 N, Fz = 20.5 N). The pure moments were applied continuously for 3.5 cycles with an angular velocity of 1°/s in flexion/extension and lateral bending, as well as 0.5°/s in axial rotation using three stepper motors (Isel 3450, Isert-electronic, Eiterfeld, Germany) with a torque of 55 Ncm and 1.8° per step. The moments were measured by a 6-DOF load cell (FT 1500/40, Schunk GmbH & Co. KG, Lauffen/Neckar, Germany), which has a measuring range of ±40 Nm, a resolution of 0.02 Nm and a measuring error of <1%. The first two cycles served for preconditioning of the specimen, while the third cycle was used for data evaluation.

**Fig 2:**
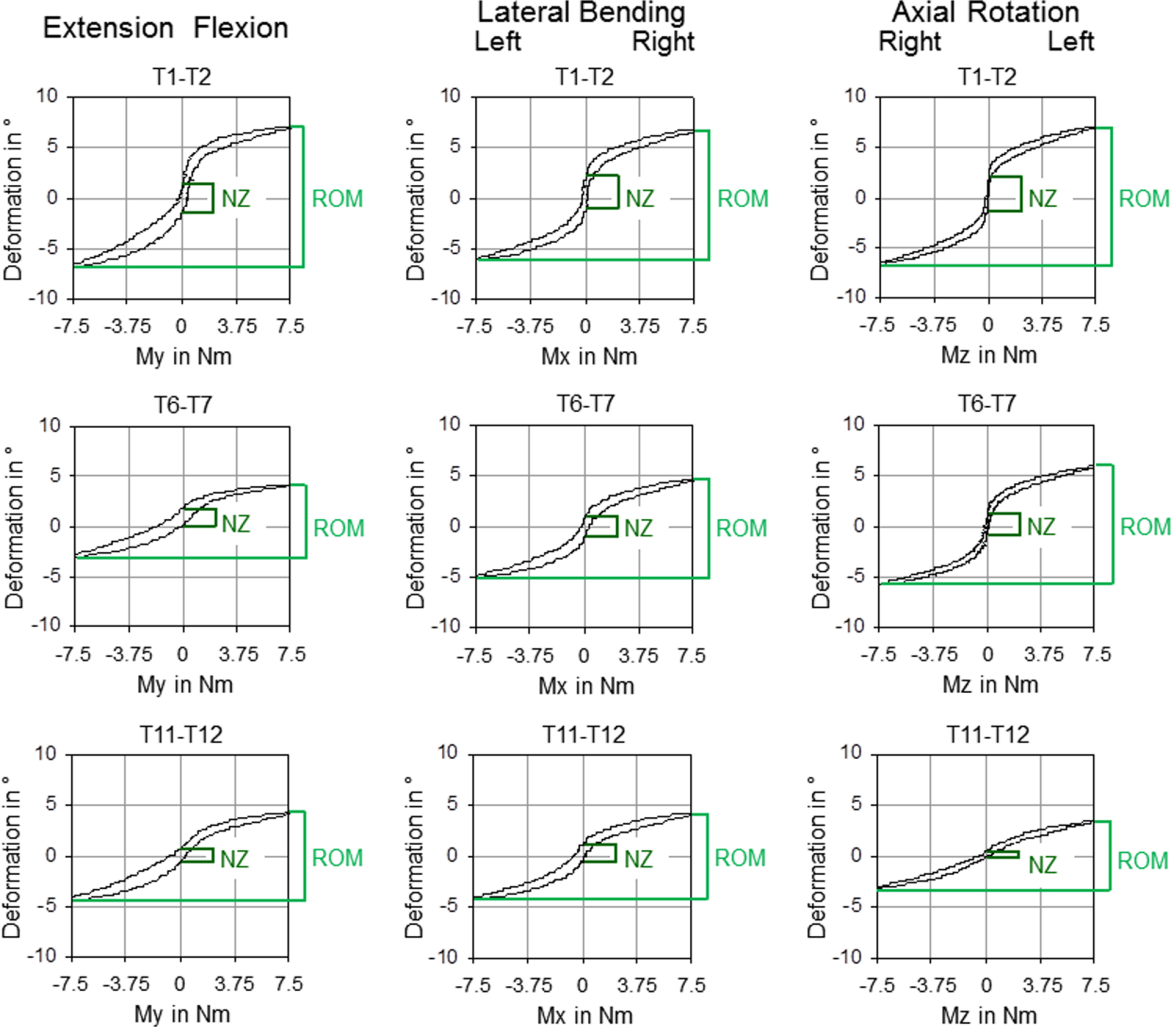
Load-deformation curves. Characteristic hysteresis curves of representative thoracic spinal motion segments of the upper, middle, and lower thoracic spine in flexion/extension, lateral bending, and axial rotation.

The resulting load-deformation curves represent the typical stiffness properties of the single motion segments (Fig 2) and were used for the determination of the biomechanical parameters range of motion (ROM) and neutral zone (NZ). The ROM describes the deformation of the specimen at the maximum load in the respective loading direction, while the NZ is the motion range of the specimen in the unloading phase (at 0 Nm) and is therefore a measure for the laxity of the motion segment [21, 22]. ROM and NZ were automatically determined using a self-programmed MATLAB routine (MathWorks Inc., Natick, USA) which fitted a polynomial function to the curve to define the midpoint of the hysteresis curve regarding the angle and measured the displacements at ±7.5 Nm (ROM) as well as at the midpoint of the connecting line between the two turning points of the polynomial function (NZ). The evaluated hysteresis curves of all experiments are depicted in the S5–S7 Dataset files.

The present in vitro study and related use of human specimens were approved by the ethical committee board of the University of Ulm, Germany (No. 302/14).

## Results

**Fig 3:**
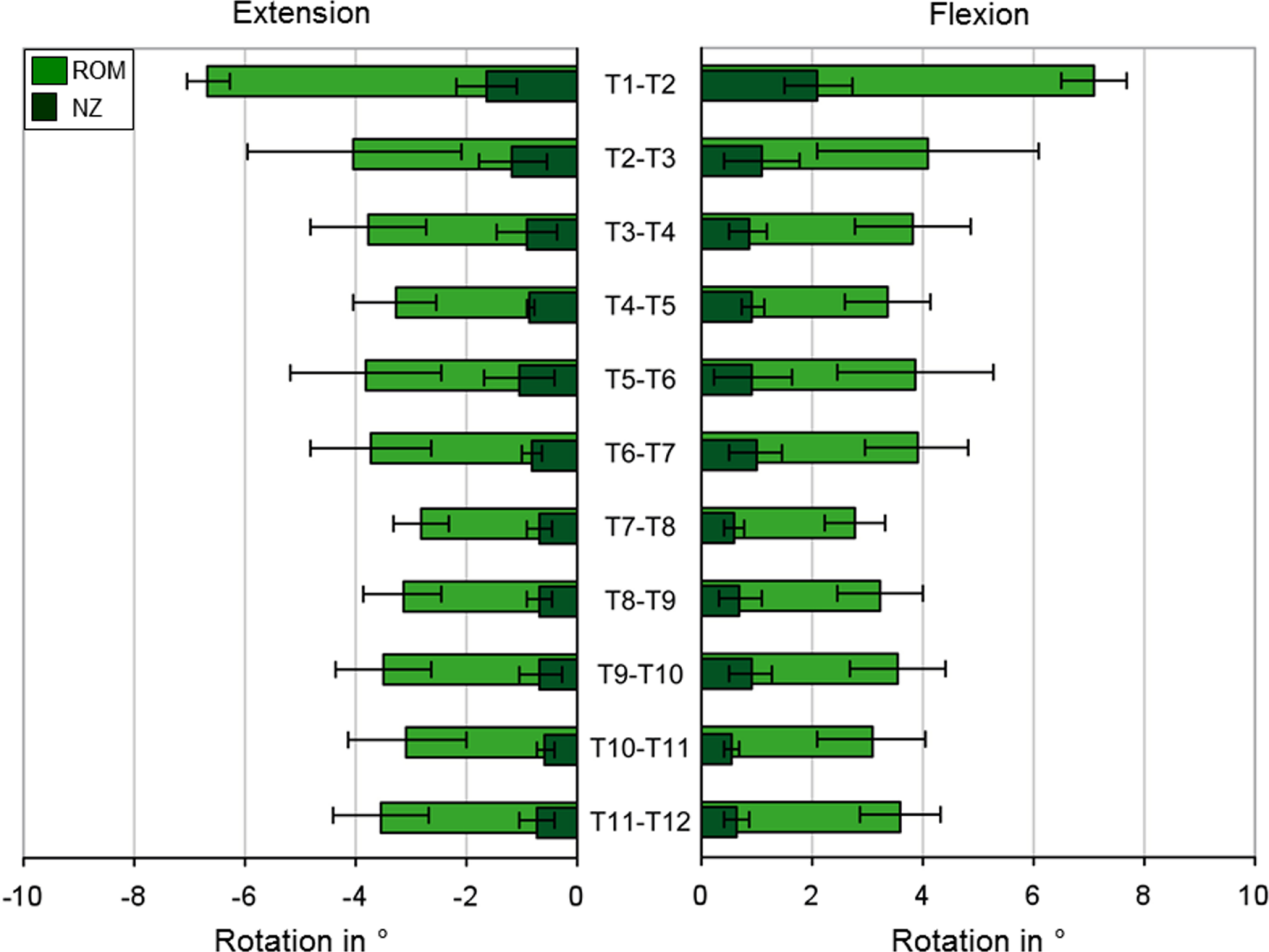
Flexion/extension. ROM and NZ at ±7.5 Nm pure moment in flexion/extension for all thoracic spinal motion segments (n = 6, except n = 7 for T4-T5 and T7-T8), represented as mean values with standard deviations.

**Fig 4:**
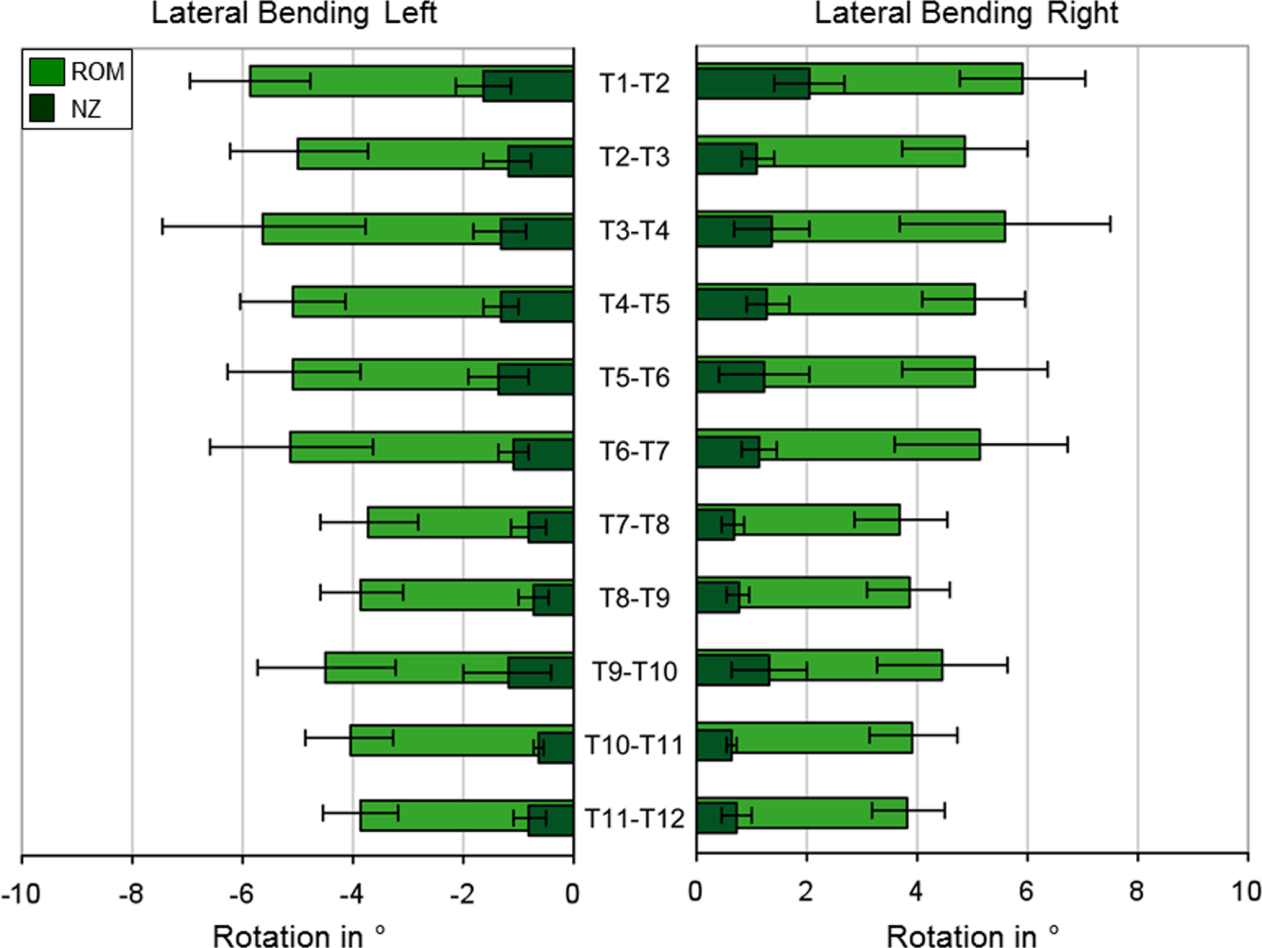
Lateral bending. ROM and NZ at ±7.5 Nm pure moment in lateral bending for all thoracic spinal motion segments (n = 6, except n = 7 for T4-T5 and T7-T8), represented as mean values with standard deviations.

**Fig 5:**
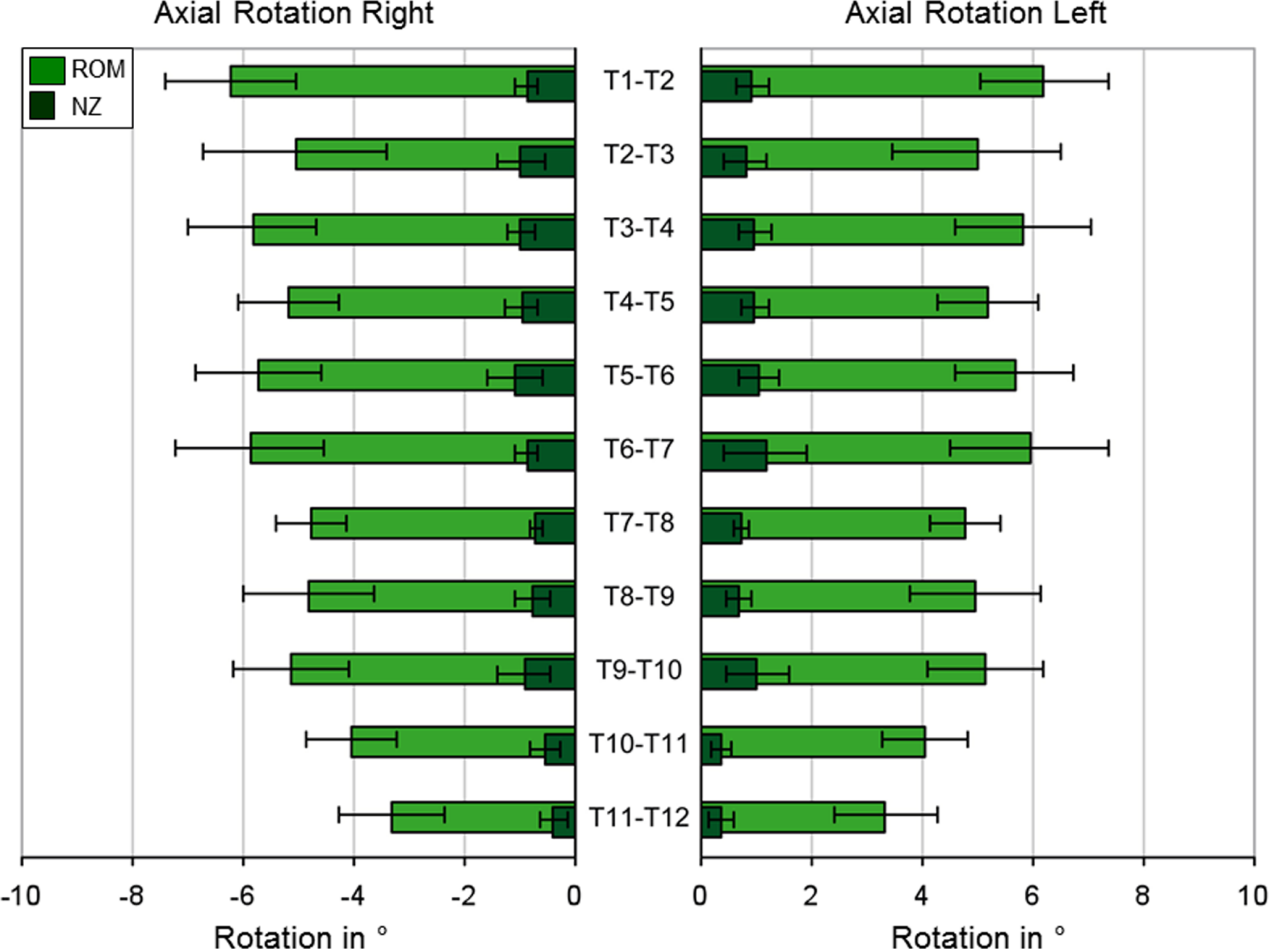
Axial rotation. ROM and NZ at ±7.5 Nm pure moment in axial rotation for all thoracic spinal motion segments (n = 6, except n = 7 for T4-T5 and T7-T8), represented as mean values with standard deviations.

The motion segment T1-T2 was found to have the highest ROM in all six loading directions, of which flexion was identified as the loading direction with the highest ROM in this specific motion segment (Figs 3–5). The upper half of the thoracic spine from T1-T2 to T6-T7 showed a higher range of motion than the lower thoracic spine from T7-T8 to T11-T12 in all loading planes. The lowest ROMs were detected in extension for all motion segments between T2-T3 and T11-T12, followed by flexion, both having ROMs equal or less than 4°, whereas the highest ROMs were generally found in axial rotation for all motion segments from T2-T3 to T10-T11 and in lateral bending for T11-T12, respectively. In axial rotation, the NZ to ROM ratio was the lowest of all three loading planes, whereas the highest NZ to ROM ratio was found in lateral bending.

In flexion/extension, the level T1-T2 showed the greatest ROM of all motion segments with a mean value of 7.1° in flexion and 6.7° in extension (Fig 3). In T2-T3, the ROM reduced to 4.1° in both flexion and extension and was lowest in the segment T7-T8 with 2.8° for both loading directions. In the upper part of the thoracic spine from T3-T4 to T6-T7 and the lower part T8-T9 to T11-T12, the mean values of the ROM ranged from 3.1° to 3.8°. The standard deviations accounted in most segments for 20–36% of the ROM, while T2-T3 showed a large standard deviation of 49% and T1-T2 a small standard deviation of 6%. The NZ was between 0.6° and 2.1°, which makes up 14–30% of the mean ROMs (S1 Dataset).

In lateral bending, the segment T1-T2 had the highest range of motion, with a mean value of 5.9° in both directions (Fig 4). The ROM of the upper and middle segments from T2-T3 to T6-T7 ranged between 4.9° and 5.6°, whereas the inferior segments exhibited a smaller range of motion between 3.8° and 4.5°. The lowest ROM was again detected in the segment T7-T8 with 3.7°. The standard deviations ranged between 17% and 34% of the mean ROMs, with the largest standard deviation in the segment T3-T4. Generally, ROM and NZ had almost symmetrical motion behavior. The NZ was between 0.7° and 2.0°, which makes up 16–35% of the mean ROMs (S1 Dataset).

In axial rotation, almost symmetrical motion behavior for the rotation to each the left and right direction was detected (Fig 5). The ROM was again highest in the first segment T1-T2 with 6.2°, followed by segment T6-T7 with 5.9°. In the upper thoracic spine from T2-T3 to T5-T6, values between 5.2° and 5.7° were measured. In the segments from T7-T8 to T9-T10 the mean ROM was about 5°, while a continuous decrease of range of motion could be detected from T9-T10 to T11-T12. The smallest ROM with a mean of 3.3° was measured in the last segment T11-T12. The standard deviations were between 18% and 31% of the mean ROMs. The NZ, with values between 0.4° and 1.2°, makes up 10–20% of the mean ROMs and was lower than in the other both motion planes (S1 Dataset).

## Discussion

The aim of the present study was to investigate the segmental range of motion and neutral zone of the thoracic spine by applying pure moments under controlled, standardized testing conditions, since the literature regarding the biomechanical properties of the single thoracic spinal motion segments is scarce and the segmental flexibility of the thoracic spine has to be determined to understand the biomechanics, to solve clinical problems, and to calibrate and validate numerical models. The present in vitro study therefore provides monosegmental ROM data for all thoracic spinal segments.

### Specimens

68 FSUs from 29 different human donors were used in this study. Complete or almost complete thoracic spines were available from nine donors and between three and five segments could be used per donor. The missing segments were replaced by individual segments from 20 other donors. At least six monosegmental specimens were tested per segment, although a higher number would have been preferable. However, due to the limited availability of human specimens, as well as ethical and financial issues, n = 6 specimens per segment is generally accepted for in vitro studies.

Specimens of young healthy donors are most appropriate to exclude variations in the biomechanical properties which are caused by degenerative remodelling processes occurring in middle age. Kettler et al. and Mimura et al. determined in their in vitro studies with lumbar specimens a decrease of ROM in flexion/extension and lateral bending with increasing degree of degeneration. A slight increase of ROM was found in axial rotation [28, 29]. Due to the unavailability of younger donors, the average age of specimens in this study was 58 years (40–80 years). Variations of donor age and thus the stage of disc degeneration could be responsible, besides specific differences in specimen morphology, for the variations within the results and could therefore affect their comparability. Since the specimens had to be randomly distributed on the single segmental level groups due to the limited availability, donor age or sex had to be disregarded. However, mean donor age and sex of the eleven segmental level groups were not substantially different between the groups, varying between 50 and 64 years and a sex ratio between 3:3 and 6:1 (Table 1).

During preparation, care was taken to preserve the costovertebral joints, since they stabilize the thoracic spine in all three loading directions [30–32]. The anterior part of the rib cage, including the sternal complex and the ribs, was excluded from the test setup to reach a high comparability between the single motion segments, although it was also found to influence the stability of the thoracic spine in former in vitro studies [33–35]. The effect of the ribs and its sternal portion on the flexibility of the single motion segments should be evaluated separately. The ligaments were left intact, since damaged or missing ligament structures were found to influence the ROM, as well as the intradiscal pressure and the relative position of the vertebral bodies [36, 37].

The specimens were checked for damage prior to preparation using conventional X-ray. CT or MRI scans, however, were not performed. The present study therefore solely provides kinematic and stiffness properties for the mechanical validation of finite element models of the thoracic spinal motion segments.

### Testing conditions

According to current recommendations for in vitro experiments, the specimens were tested with 3.5 loading cycles, of which the third loading cycle was evaluated [21, 22]. This procedure has become the state of the art for biomechanical flexibility testing, because the first two loading cycles are used for preconditioning to reduce viscoelastic effects [22, 26]. Moreover, the monosegmental ROMs were determined by using pure moments of ±7.5 Nm to allow direct comparison to ROM data in the literature for the lumbar spine, although ±5 Nm are recommended for thoracic spinal flexibility testing [38]. Since there was no visible change in range of motion, hysteresis, and elastic stiffness during all loading cycles, pure moments of ±7.5 Nm were considered as acceptable for testing (S1 Dataset).

An axial preload was omitted in the present study, although it is recommended for spinal in vitro testing [39, 40]. Axial preload may reduce the segmental mobility and effect the kinematic response, wherefore it was used in previous studies to simulate the experimental motion of the spine as physiologically as possible [41–43].

Another limitation of the present study represents the manual alignment of the specimens in the spine tester, which could have led to slight off axis loads in flexion/extension and lateral bending. However, it was tried to compensate the possible angle offsets by automatic determination of each hysteresis curve midpoint.

Care was also taken not to exceed the testing period of 20 hours during preparation and testing for each specimen, since the biomechanical properties of the tested specimens will change and autolytic processes will start [21].

### Biomechanical interpretation of the results

Specific anatomical properties influence the range of motion of the single spinal regions. The cross-sectional areas of the discs of the upper thoracic spine, for example, are relatively small compared to those in the lower thoracic spine and increase inferiorly, whereas the disc heights are approximately the same in the upper and lower thoracic spine [44], leading to a higher ROM, given the same amount of pure moments for all segmental levels in our in vitro study, in the upper thoracic spinal motion segments because of the lower moment of inertia of area. In the present study, the first segment T1-T2 exhibited the highest flexibility of all segments in all loading directions, indicating similar range of motion characteristics as the cervicothoracic transition, whereas the range of motion tended to decrease in inferior direction towards the lumbar spine in all six loading directions (Figs 3–5).

Another anatomical characteristic of the thoracic spine is provided by the different positions of the costovertebral joints. While these joints are positioned each between the single motion segments at the level of the discs for all segments from T1-T2 to T9-T10, potentially having a stabilizing effect together with the costotransverse joints and their ligament structures, the costovertebral joints of the two inferior pairs of ribs are located on the middle of T11 and T12, respectively. The costovertebral joints therefore have no potential stabilizing effect on the motion segments T10-T11 and T11-T12. However, these two motion segments showed an equal or lower ROM compared to the other motion segments in the present study, indicating a larger stabilizing effect of the discs, ligaments, facet joint capsules, and facet joint orientations in these motion segments. Moreover, a stabilizing effect of the costovertebral joints was only determined in combination with a radical discectomy in previous in vitro studies [30, 45].

The facet joints of the thoracic spine show a specific morphology because of their three-dimensional orientation, which leads to a different motion behavior compared to the cervical and the lumbar spine. In the inferior direction towards the lumbar spine, the facet joints exhibit increasing tilt angles in the sagittal plane and slightly in the transversal plane [46], limiting the range of motion, especially in flexion/extension and slightly in axial rotation. The variation of the facet joint orientations within the thoracic spine can be high due to interindividual differences, but in general, the tilt angles increase gradually, whereby the tilt angles in the transversal plane end in the thoracolumbar transition zone [26, 47]. Also the thoracic spinal ligaments have a strong stabilizing effect, since they are thicker than those in the cervical and lumbar spine [26, 48]. Together with the anterior part of the rib cage, the anterior and posterior longitudinal ligaments prevent hyperflexion and -extension [49].

### Literature comparison

Few in vitro studies have investigated the flexibility of the thoracic spine. Furthermore, the previously existing results are only partially comparable due to varying experimental setups and loading conditions. By means of new basic in vitro data of the thoracic spinal flexibility, evaluated using now widely accepted recommendations for in vitro testing of spinal segments [21, 22], a better interpretation of the already published data should be possible. In addition, a comprehensive database could provide a basis for the development of new finite element models or multi-body systems of the healthy human thoracic spine.

**Fig 6:**
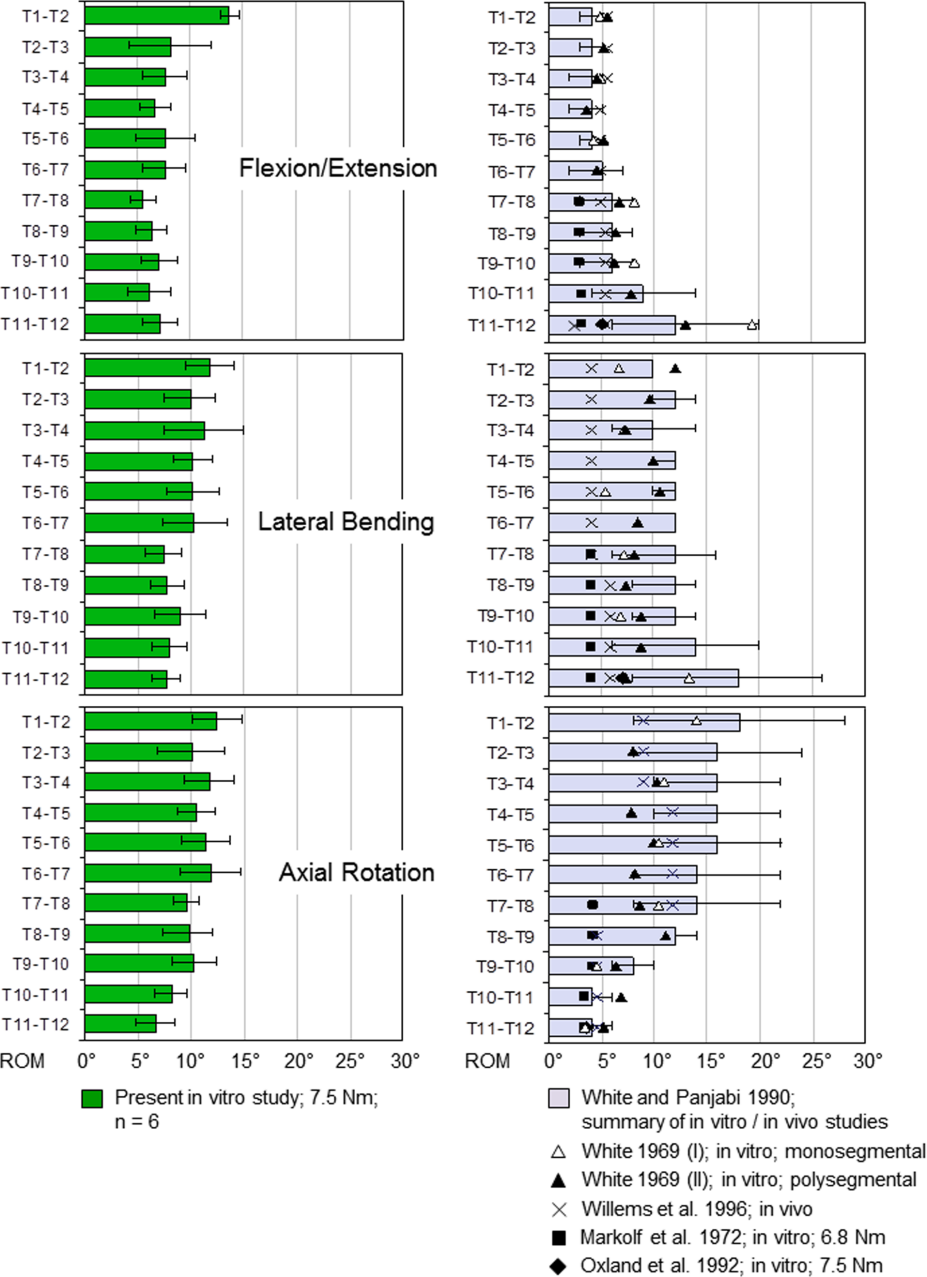
Literature comparison. Comparison of the ROM data evaluated in the present study, represented as mean values with standard deviations of the full ROM in each loading plane, with data extracted from the literature. The data of White and Panjabi [26] are represented as mean values with value ranges.

The subsequent literature overview compares the results of the present study and previously published results of in vitro and in vivo studies (Fig 6). The flexibility tests of the present study were performed in a well-established spine tester [27]. One specific feature of this device is the application of pure moments, which ensures that the load is applied precisely and reproducibly on the whole tested spinal segment in each motion plane. Similar methods for in vitro testing of spinal segments with pure moments were found in the literature for the experiments of Panjabi et al., Markolf et al., and Oxland et al. [23–25]. The study, which is closest to the present study because of its comprehensiveness, has been described by White [20], who applied eccentric loads in his two-dimensional and three-dimensional analysis of flexion/extension and lateral bending motions. It should be noted, however, that White and Markolf et al. used an experimental setup, in which the upper vertebra exhibited a limited mobility in the remaining five motion directions [20, 24], which could have limited flexibility in the main loading direction.

A literature overview was published by White and Panjabi [26], where ROM data of several in vitro and in vivo studies, including different test setups and load applications, were summarized. Therefore, these data allow comparisons with other data only in a limited extent.

Moreover, the in vivo study of Willems et al. was performed using the polysegmental sections T1-T4, T4-T8 and T8-T12 [50]; for better comparability, the values have been divided according to the number of segmental portions. However, the flexibility of the individual spinal segments is not known. When comparing in vitro and in vivo data, it should also be noted that no standardized torque limits exist during in vivo measurements. Furthermore, other effects, such as the rib cage, the muscles, the intraabdominal and intrathoracic pressure, as well as individual pain sensations, have an influence on the flexibility of the spinal segments.

In general, differences regarding segmental flexibility between the three loading planes were not as significant as those described in literature (Fig 6). In flexion/extension, the data of the present study show the highest ROM in the first segment T1-T2. However, there is an inverse trend of the results compared to the summary from White and Panjabi [26] with the highest ROM at T11-T12, whereas the results of Markolf et al., Oxland et al., and Willems et al. show a similar motion behavior for the lower thoracic segments [24, 25, 50]. Besides, the data of White and Panjabi exhibited high value ranges in these two lower segments, which can probably be explained by the high interindividual variations regarding the facet joint orientations in the thoracolumbar transition zone from T11-T12 to L1-L2 [47]. Morita et al. [51] evaluated similarities in their in vivo analysis of flexion/extension ROM, but just about half as high ROM values as Willems et al. [50], which is probably due to their test setup, where the ROMs were measured by CT scans in lying position.

In lateral bending, the ROM data of the present study are quite comparable with literature data, especially regarding the upper half of the thoracic spine from T1-T2 to T6-T7. However, the increase of ROM in T10-T11 and T11-T12 in the summary of White and Panjabi [26], including the high value ranges, should be mentioned.

In axial rotation, the ROM values of the present study and their progression are quite comparable with the data in the literature, where in general the same progression of ROM values was detected. In the in vivo study of Gregersen and Lucas [52], using Steinmann pins, average ROMs of 7° for all thoracic spinal motion segments were detected in each standing and sitting position, with a substantially higher ROM in the segment T1-T2. In contrast, Fujimori et al. [53] evaluated in their in vivo study ROMs between 2° and 5° for all thoracic spinal motion segments and increasing ROMs from T1-T2 to T9-T10, which is also probably due to their test setup, where they analyzed the thoracic spinal range of motion using CT Scans in lying position.

## Conclusions

The literature regarding the biomechanical properties of the single thoracic spinal motion segments is scarce. Due to the limited available data of the few previous studies with partially contradictory data and different measurement methods, the segmental flexibility of the whole thoracic spine has to be determined to understand the biomechanics, to solve clinical problems, and to calibrate and validate numerical models of the thoracic spine.

The present study showed that in flexion/extension, the thoracic spinal segments have the lowest range of motion in the spinal section from T2-T3 to T11-T12, but the highest range of motion in T1-T2, of all three loading planes. In lateral bending, the upper half of the thoracic spine from T1-T2 to T6-T7 showed generally a higher range of motion than the lower half from T7-T8 to T11-T12. The highest range of motion was observed in the upper and middle segments from T2-T3 to T9-T10 in axial rotation, where also a decrease in range of motion in the lower thoracic segments was observed.

In flexion/extension as well as in lateral bending, no increase of range of motion in the lower thoracic segments could be detected in the present study, which partially contrasts with former in vitro studies. It is also remarkable that a higher range of motion was determined for the upper thoracic spinal segments in all loading directions.

The data of the present study could be used for the validation of numerical models and the design of further in vitro studies of the thoracic spine, the verification of applicability of animal models, as well as the interpretation of already published human in vitro data.

## Supporting information

S1 Dataset Raw data. The data including all ROM and NZ values in all six loading directions of the present in vitro study are summarized. Additionally, neutral zone stiffness (NZS), elastic zone stiffness (EZS), and hysteresis area are listed.
(XLSX)

S2 Dataset Load-displacement raw data T1-T5. The load-displacement raw data of all experiments regarding the segmental levels T1-T2, T2-T3, T3-T4, and T4-T5 including all 3.5 loading cycles are listed.
(XLSX)

S3 Dataset Load-displacement raw data T5-T9. The load-displacement raw data of all experiments regarding the segmental levels T5-T6, T6-T7, T7-T8, and T8-T9 including all 3.5 loading cycles are listed.
(XLSX)

S4 Dataset Load-displacement raw data T9-T12. The load-displacement raw data of all experiments regarding the segmental levels T9-T10, T10-T11, and T11-T12 including all 3.5 loading cycles are listed.
(XLSX)

S5 Dataset Hysteresis curves T1-T5. The hysteresis curves of the third loading cycle of all experiments regarding the segmental levels T1-T2, T2-T3, T3-T4, and T4-T5 are depicted.
(XLSX)

S6 Dataset Hysteresis curves T5-T9. The hysteresis curves of the third loading cycle of all experiments regarding the segmental levels T5-T6, T6-T7, T7-T8, and T8-T9 are depicted.
(XLSX)

S7 Dataset Hysteresis curves T9-T12. The hysteresis curves of the third loading cycle of all experiments regarding the segmental levels T9-T10, T10-T11, and T11-T12 are depicted.
(XLSX)
